# Multi-hazard spatial modeling via ensembles of machine learning and meta-heuristic techniques

**DOI:** 10.1038/s41598-022-05364-y

**Published:** 2022-01-27

**Authors:** Mojgan Bordbar, Hossein Aghamohammadi, Hamid Reza Pourghasemi, Zahra Azizi

**Affiliations:** 1 Department of Remote Sensing and GIS, Faculty of Natural Resources and Environment, Science and Research Branch, Islamic Azad University, Tehran, Iran; 2 Department of Natural Resources and Environmental Engineering, College of Agriculture, Shiraz University, Shiraz, Iran

**Keywords:** Environmental sciences, Natural hazards

## Abstract

Considering the large number of natural disasters on the planet, many areas in the world are at risk of these hazards; therefore, providing an integrated map as a guide map for multiple natural hazards can be applied to save human lives and reduce financial losses. This study designed a multi-hazard map for three important hazards (earthquakes, floods, and landslides) to identify endangered areas in Kermanshah province located in western Iran using ensemble SWARA-ANFIS-PSO and SWARA-ANFIS-GWO models. In the first step, flood and landslide inventory maps were generated to identify at-risk areas. Then, the occurrence places for each hazard were divided into two groups for training susceptibility models (70%) and testing the models applied (30%). Factors affecting these hazards, including altitude, slope aspect, slope degree, plan curvature, distance to rivers, distance to roads, distance to the faults, rainfall, lithology, and land use, were used to generate susceptibility maps. The SWARA method was used to weigh the subclasses of the influencing factors in floods and landslides. In addition, a peak ground acceleration (PGA) map was generated to investigate earthquakes in the study area. In the next step, the ANFIS machine learning algorithm was used in combination with PSO and GWO meta-heuristic algorithms to train the data, and SWARA-ANFIS-PSO and SWARA-ANFIS-GWO susceptibility maps were separately generated for flood and landslide hazards. The predictive ability of the implemented models was validated using the receiver operating characteristics (ROC), root mean square error (RMSE), and mean square error (MSE) methods. The results showed that the SWARA-ANFIS-PSO ensemble model had the best performance in generating flood susceptibility maps with ROC = 0.936, RMS = 0.346, and MSE = 0.120. Furthermore, this model showed excellent results (ROC = 0.894, RMS = 0.410, and MSE = 0.168) for generating a landslide map. Finally, the best maps and PGA map were combined, and a multi-hazard map (MHM) was obtained for Kermanshah Province. This map can be used by managers and planners as a practical guide for sustainable development.

## Introduction

One of the main environmental problems that have affected different countries in recent years is natural hazards^1^. According to global statistics, 40% of economic and social damage is caused by natural hazards^2^. A hazard can be described as a potentially destructive physical event with the possibility of human death or injury, socio-economic losses, or destruction of the natural environment^3^. Natural hazards include floods, earthquakes, landslides, tsunamis, volcanoes, and erosion^4^. In one region, one of these hazards or even several different hazards occur depending on the origin and effects^3^. Therefore, it is of great importance to study many of these hazards together to achieve integrated management in an area. The multi-hazard was first proposed by the United Nations Environment Program (Agenda 21) to manage urban areas susceptible to natural disasters^5^. Today, economic and human losses due to these hazards are rapidly increasing worldwide^6^. Iran is also one of the regions exposed to various natural disasters that have caused significant financial and human losses. Therefore, providing a multi-hazard map (MHM) can be an effective tool for managers and planners to reduce economic losses and casualties. In this regard, using spatial methods and considering several hazards in an area, it is possible to identify at-risk areas^7^. Remote sensing (RS) and Geographic Information System (GIS) are effective and quick tools for identifying areas susceptible to hazards by collecting, storing, combining, manipulating, retrieving, analyzing, and displaying information^8^.

According to the statistics reported, earthquakes are considered as one of the most catastrophic and unpredictable natural hazards, ranking second in terms of the effects and risks of human life among all types of natural disasters^9^. Iran, which is classified as an earthquake-prone country, has experienced 18 earthquakes with a magnitude of more than 7 Richter in the last 90 years with severe economic and social damage and high mortality^10^. Therefore, it is necessary to conduct research on earthquakes to determine probable areas of earthquakes. Floods are also high-risk natural disasters, and one of the main reasons for their occurrence is land use change. Moreover, the removal of vegetation and soil, formation of drainage networks, and increased surface runoff and floods^11^. This hazard has affected the lives of more than 20,000 people annually and has caused considerable damage^12^. Therefore, it is of importance to generate a susceptibility map for regions prone to floods^8,13–15^. Another important natural hazard that causes great damage to the mountainous areas is landslides^16^. Factors affecting landslides depend on the features of the region, including natural features such as extreme rainfall, snowmelt, and human-made factors^17^. Recently, landslides occurred in Iran have been mostly due to land tectonics, climatic, vegetative, and human activities^17^. Therefore, it is necessary to generate a susceptibility map of this hazard, which has been studied in many areas of the world^18–22^.

To date, various methods have been applied to generate hazard susceptibility maps. Some of these methods include multivariate statistical methods such as logistic regression (LR)^23^, the analytical hierarchy process (AHP)^24^, multi-criteria evaluation^25^ and soft computing models, such as decision trees^26^, random forest (RF)^27^, artificial neural networks (ANNs)^28,29^, fuzzy logic^30,31^, support vector machine method (SVM)^32^, and adaptive neuro-fuzzy inference system algorithm (ANFIS)^33^ have been applied to assess various hazards. In addition, a combination of machine learning and meta-heuristic learning algorithms have been developed with successful results for studying natural hazards^34^. Meta-heuristic algorithms to implement learning machines improve and increase the predictability of models^35^. In this study, the ANFIS machine learning algorithm was selected because it uses both ANN and fuzzy logic. To find the optimum weight of the parameters of this learning algorithm and increase its capability, grey wolf optimizer (GWO) and particle swarm optimization (PSO) algorithms were selected^36^. Moreover, the PSO and GWO techniques were employed to overcome the limitations of the ANFIS method via optimization.

In recent years, the simultaneous study of several natural hazards has attracted the attention of many researchers worldwide. Examples include landslides and earthquakes in India^37^, landslides, floods, erosions, and earthquakes in Greece^38^, climatic hazards in the United States^39^, climatic hazards in Chile^40^, and avalanches, rock falls, and floods in Iran^41^. Different characteristics of each hazard and interactions between hazards may provoke each other. This means that the occurrence of one hazard can cause another hazard. For example, earthquakes can cause landslides or even occur simultaneously^42^. In addition, landslides and floods are closely related to each other and are often accompanied by heavy or prolonged rainfall^43^. Heavy rains also cause sudden floods, resulting in soil erosion and landslides occurrence^44^.

The earthquake occurred on Sunday evening, November 12, 2017 near Ezgeleh in Kermanshah province (7.3 Richter), is one of the largest earthquakes in recent years, in which 600 to 700 people died and nearly 10,000 people injured^45^. In April 2019, one of the great floods in recent years occurred in this province and caused considerable economic damage (https://kurdpress.com). Moreover, this province is one of the mountainous areas of the Zagros Mountains in Iran, which is at risk of landslides. These three natural hazards, which have caused considerable human and financial losses in this province, have not yet been studied together in Kermanshah province. Therefore, this study investigates these three natural hazards (earthquakes, floods, and landslides) in individuals and in combination with each other in order to determine the areas prone to these natural hazards in Kermanshah Province. The SWARA-ANFIS-PSO ensemble model was used for the first time in multi-hazard studies to prepare a multi-hazard susceptibility map (MHSM). The results were then compared with those of the SWARA-ANFIS-GWO ensemble model.

## Results and discussion

Multicollinearity analysis of influencing factors. Multicollinearity test was used in the present study to investigate the correlation between factors affecting natural hazards of floods and landslides. Inflation coefficient of variance (VIF) and Tolerance indicate the effect of collinearity between the factors. If there is linearity between the factors, the factor should be removed from the modeling. The results of this method for the hazard of landslides showed that factor “Lithology” has the highest VIF value (1.696) and the lowest tolerance (0.590). The results of this method for the hazard of floods showed that the factor “lithology” has the highest VIF value (2.068) and the lowest tolerance (0.483). The results of the analysis showed that all the factors used in the assessment of flood and landslide hazards can be applied in modeling. In other words, there is no multi-collinearity between the factors used (Table 1). In addition, the commonly used information gain ratio (IGR) method was used to determine the importance of the influencing factors used and it was implemented considering the significant effect on the accuracy of the estimation. The results of the information gain ratio (IGR) method showed that the lithology factor has the highest impact on Kermanshah’s landslide events (0.56), followed by slope degree (0.52), altitude (0.24), distance to road (0.17), land use (0.11), aspect (0.046), distance to fault (0.044), plan curvature (0.041), and distance to river (0.02). Moreover, based on the IGR method the altitude factor (0.73) is the most important factor for flood events in the study area, followed by lithology (0.44), rainfall (0.28), slope degree (0.22), distance to river (0.17), plan curvature (0.10), land cover (0.05), and aspect (0.03) (Table 2).

**Table 1:** Multicollinearity analysis.

Influencing factor	Landslide		Flood	
	Tolerance	VIF	Tolerance	VIF
Altitude	0.668	1.498	0.616	1.622
Slope degree	0.689	1.451	0.679	1.474
Slope aspect	0.928	1.078	0.951	1.052
Plan curvature	0.868	1.153	0.790	1.266
Distance to roads	0.909	1.100	–	–
Distance to faults	0.837	1.194	–	–
Distance to rivers	0.921	1.086	0.813	1.230
Lithology	0.590	1.696	0.483	2.068
Land use	0.915	1.093	0.848	1.179
Rainfall	–	–	0.660	1.515

**Table 2:** Importance of natural hazards influencing factors using the information gain ratio (IGR) method.

Flood influencing factor	Altitude	Lithology	Rainfall	Slope degree	Distance to river	Plan curvature	Land cover	Aspect	–
IGR value	0.73	0.44	0.28	0.22	0.17	0.10	0.05	0.03	–

Landslide influencing factor	Lithology	Slope degree	Altitude	Distance to road	Land use	Aspect	Distance to fault	Plan curvature	Distance to river
IGR value	0.56	0.52	0.24	0.17	0.11	0.046	0.044	0.041	0.02

### The step-wise weight assessment ratio analysis (SWARA) model

The SWARA model was applied to weigh each sub-factor of each influencing factor. The final weights of each class of influencing factors were standardized to be between 1 and 0. The SWARA values are presented in Table 3. The analysis for the factor of altitude showed that class 1603–1810 m (0.42) were the most prone to landslides, and class 512–828 m (0.37) were the most prone to floods. For the slope degree, class 0–5 had the highest SWARA value (0.34) for floods, while class 5–10 had the maximum value (0.41) for landslides. For the slope aspect, the highest values of SWARA were obtained for flat areas (0.22) and north areas (0.47), with respect to floods and landslides, respectively. For the plan curvature, the maximum SWARA for flood hazard was obtained for the flat region (0.39), while the greatest SWARA value was obtained for the landslide hazard for the concave class (0.42). In the case of distance to river, for both cases, the class 0–365 m showed the highest value (0.31 and 0.37) for flood and landslide hazards, respectively. For rainfall, the highest weight of SWARA was in the class 270–376 mm (0.35) for floods in Kermanshah Province. The Plbk class of lithology, with a SWARA value of 0.30, showed the highest probability of flooding, while the KEpd-gu class with a SWARA weight of 0.43 indicated the highest probability of landslide. Regarding land use, the highest SWARA was obtained for water areas (0.41), while the greatest value (0.36) was found for urban and residential areas with respect to floods and landslides, respectively. For the factor of distance to the faults, the greatest weight of SWARA (0.33) was obtained for the class more than 5000 m for landslides. According to the results for distance to road, the maximum value of SWARA (0.44) was obtained for the class more than 5000 m for landslide hazard. According to previous studies, the areas with the lowest altitude, flat regions, lowest slope degree, nearest to the river, and water use are the most prone to flooding^46^. In addition, Dai and Lee.^47^ demonstrated that the probability of landslides is high at intermediate altitudes. Moreover, urban and residential areas are more prone to landslides^22^. The present study confirmed these findings.

**Table 3:** SWARA weight for each influencing factor.

Factor	class	SWARA weight flood	SWARA weight landslide
Altitude (m)	0–512	0.19	0.02
	512–828	0.37	0.02
	828–1162	0.12	0.03
	1162–1416	0.08	0.05
	1416–1603	0.06	0.13
	1603–1810	0.05	0.42
	1810–2056	0.05	0.23
	2056–2430	0.05	0.02
	2430–3372	0.05	0.08
Aspect	Flat	0.22	0.01
	North	0.09	0.47
	North-East	0.08	0.01
	East	0.12	0.01
	South-East	0.08	0.02
	South	0.08	0.13
	South-West	0.08	0.07
	West	0.15	0.04
	North-West	0.10	0.24
Distance to river (m)	0–365	0.31	0.37
	365–758	0.14	0.21
	758–1179	0.20	0.09
	1179–1638	0.10	0.06
	1638–2150	0.06	0.04
	2150–2757	0.08	0.14
	2757–3525	0.04	0.03
	3525–4616	0.04	0.03
	4616–8105	0.04	0.03
Rainfall (mm)	270–376	0.35	–
	376–439	0.20	–
	439–490	0.14	–
	490–553	0.10	–
	553–679	0.10	–
	679–889	0.11	–
Land use	Urban and residential	0.23	0.36
	Water	0.41	0.09
	Forest	0.07	0.09
	Outcrop	0.07	0.22
	Farm land	0.14	0.14
	Range land	0.09	0.09
Lithology	Ekn	0.05	0.08
	EMas-sb	0.02	0.00
	K1bl	0.04	0.00
	Kbgp	0.01	0.03
	KEpd-gu	0.03	0.43
	Klsol	0.00	0.00
	KPeam	0.01	0.01
	Mgs	0.18	0.00
	MuPlaj	0.12	0.01
	Ogb	0.00	0.24
	OMas	0.07	0.01
	PeEtz	0.02	0.00
	pd	0.00	0.02
	Plbk	0.30	0.04
	Qft1	0.01	0.00
	Qft2	0.09	0.00
	TRKubl	0.02	0.00
	TRKurl	0.03	0.13
	Others	0.00	0.00
Slope (^0^)	0–5	0.34	0.11
	5–10	0.22	0.41
	10–15	0.16	0.14
	15–25	0.14	0.22
	25 <	0.14	0.11
Plan curvature (100/m)	Concave	0.31	0.42
	Flat	0.39	0.29
	Convex	0.31	0.29
Distance to fault (m)	< 1000	–	0.15
	1000–2000	–	0.12
	2000–3000	–	0.20
	3000–4000	–	0.10
	4000–5000	–	0.10
	> 5000	–	0.33
Distance to road (m)	< 1000	–	0.24
	1000–2000	–	0.06
	2000–3000	–	0.08
	3000–4000	–	0.06
	4000–5000	–	0.13
	> 5000	–	0.44

### Natural hazard susceptibility maps (NHSMs)

**Figure 1:**
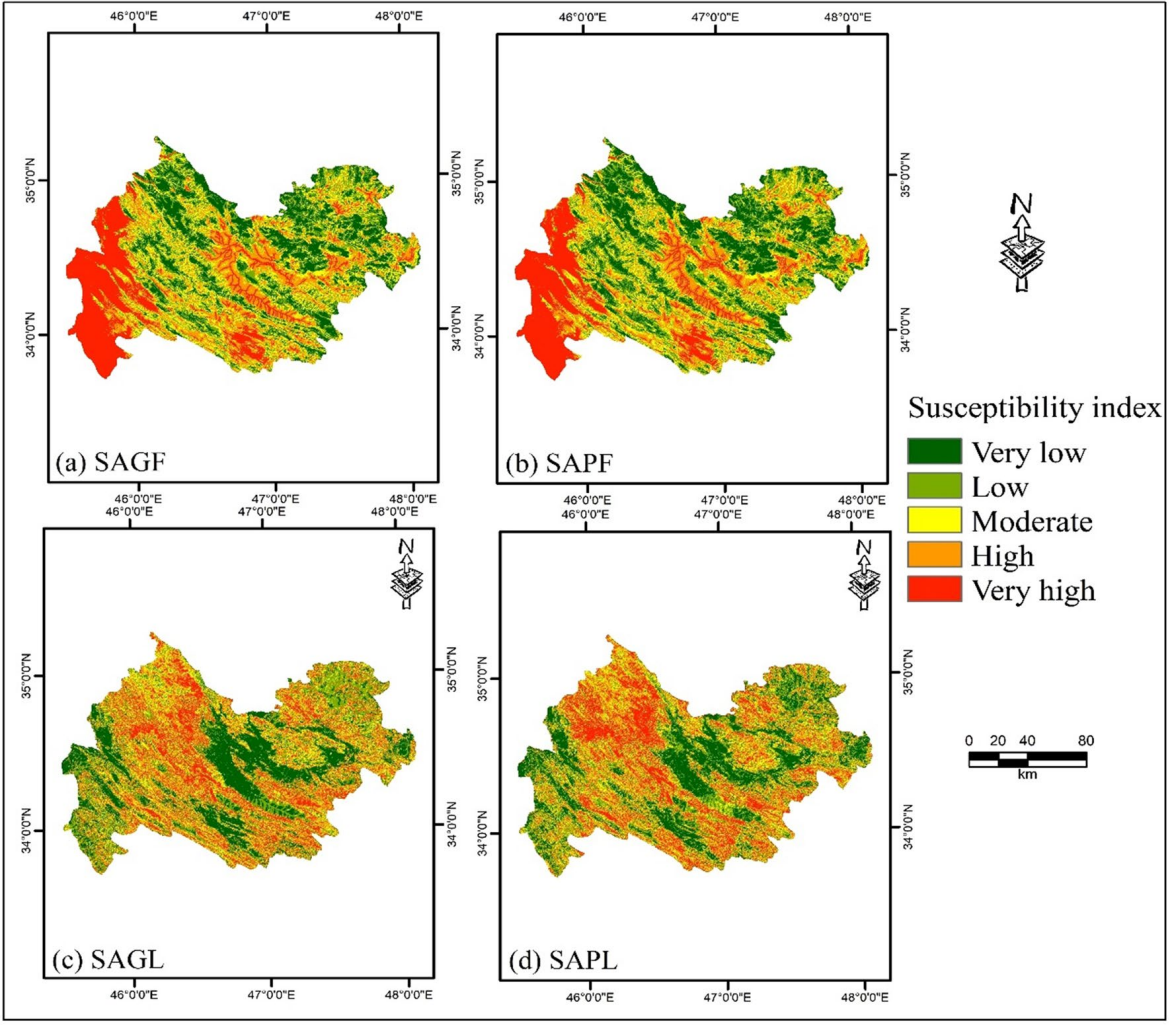
Natural hazard susceptibility maps generated by (**a**) SAGF, (**b**) SAPF, (**c**) SAGL, and (**d**) SAPL using ArcGIS 10.3.1 software (https://www.esri.com).

In this work, the SWARA model was used to weight influencing factors. ANFIS-GWO and ANFIS-PSO ensemble models were used to train the dataset to generate NHSMs for flood and landslide hazards. Flood hazard susceptibility (FHS) maps were separately generated using the SWARA-ANFIS-GWO-Flood (SAGF) and SWARA-ANFIS-PSO-Flood (SAPF) ensemble models (Fig. 1a,b). These maps revealed that the western part of Kermanshah Province had the highest susceptibility to flooding. Landslide hazard susceptibility (LHS) maps were separately produced from the SWARA-ANFIS-GWO-Landslide (SAGL) and SWARA-ANFIS-PSO-Landslide (SAPL) ensemble models (Fig. 1c,d). These maps show that the northern part of the area exhibits the highest susceptibility to landslide events. These NHSMs were then divided into five classes ranging from very low to very high susceptibility using quantile classification scheme.

### Validation of hazard maps

**Figure 2:**
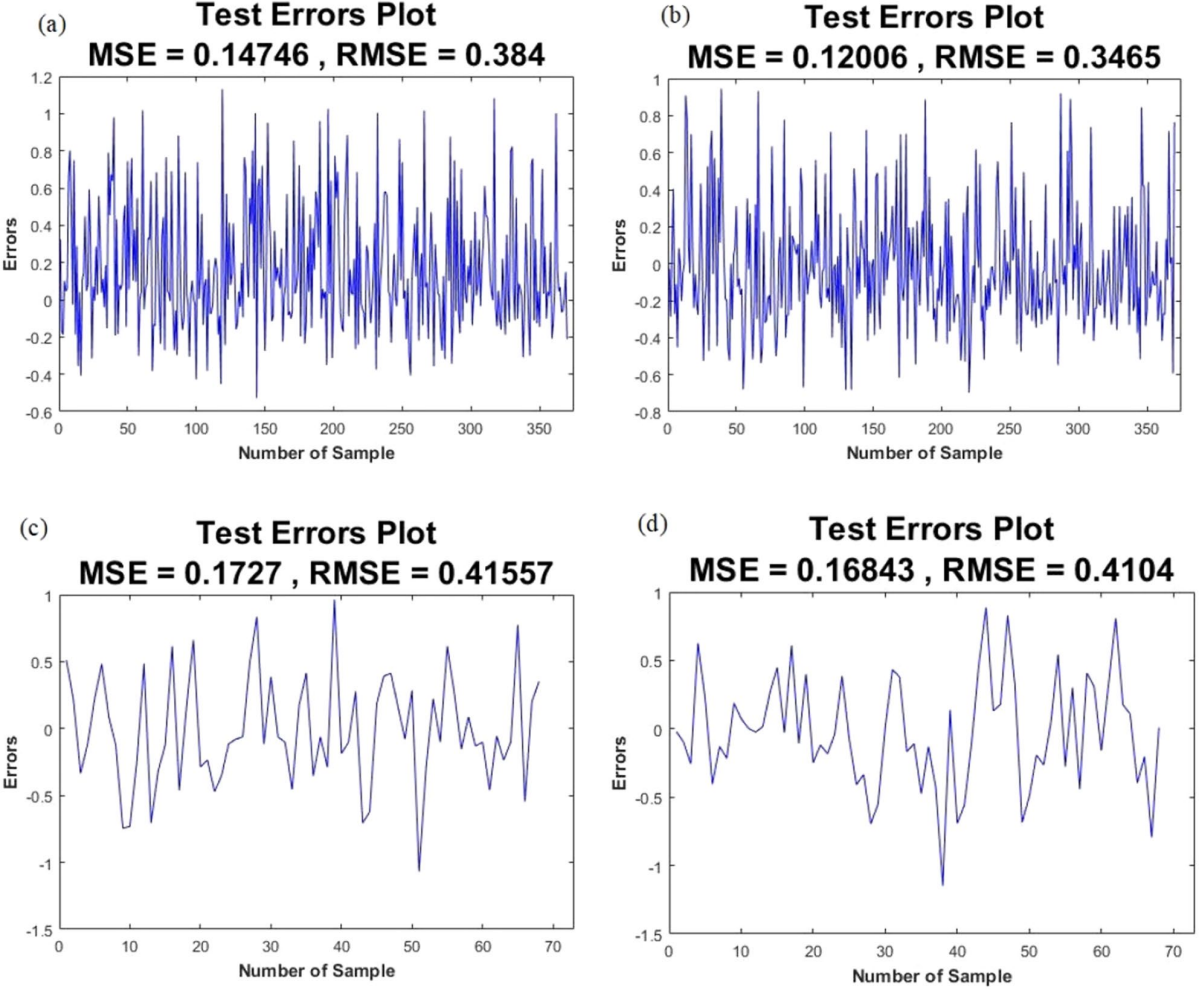
MSE and RMSE values of testing step for: (**a**) SAGF, (**b**) SAPF, (**c**) SAGL, (**d**) SAPL.

**Figure 3:**
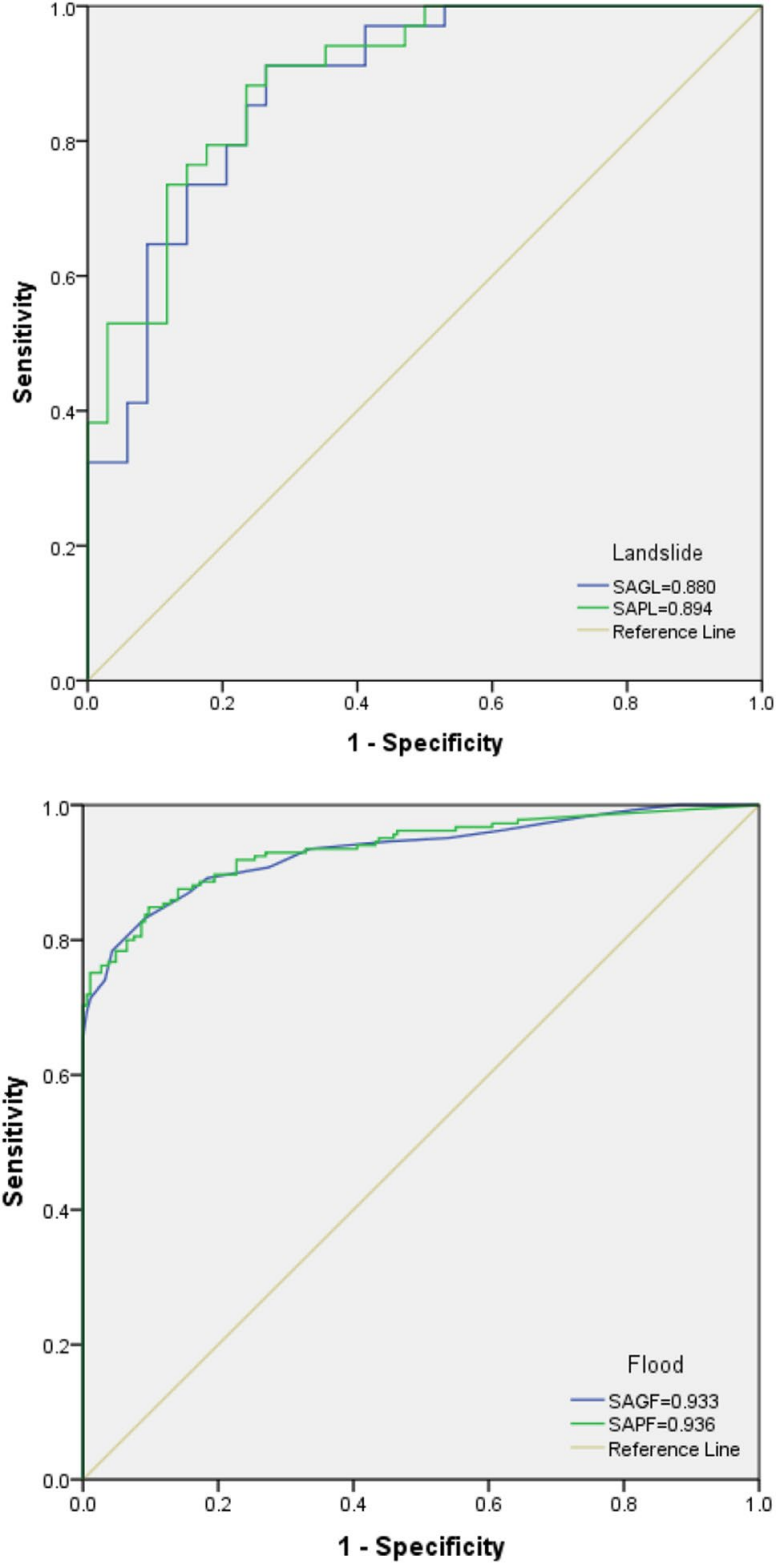
Validation of flood and landslide susceptibility maps using the ROC curve.

The power of the applied models was tested using the ROC curve, RMSE, and MSE. For flood hazard, the results illustrated that the SAPF ensemble model had the maximum AUC value (0.936) and the least error (RMSE = 0.346 and MSE = 0.120) in the testing step, followed by the SAGF ensemble model (AUC = 0.933, RMSE = 0.384, and MSE = 0.147). For landslide hazard, the results illustrated that the SAPL ensemble model had the highest prediction power.AUC = 0.894, RMASE = 0.410, and MSE = 0.168), followed by the SAGL ensemble model (AUC = 0.880, RMASE = 0.415, and MSE = 0.172) (Figs. 2, 3). Finally, the SAPF and SAPL ensemble models were selected as the suitable models for flood and landslide susceptibility assessments, respectively (Table 4).

**Table 4:** ROC, RMSE, and MSE values for each model.

Model	ROC	MSE	RMSE
SAGF	0.933	0.147	0.384
SAPF	0.936	0.120	0.346
SAGL	0.880	0.172	0.415
SAPL	0.894	0.168	0.410

### Earthquake hazard map

**Figure 4:**
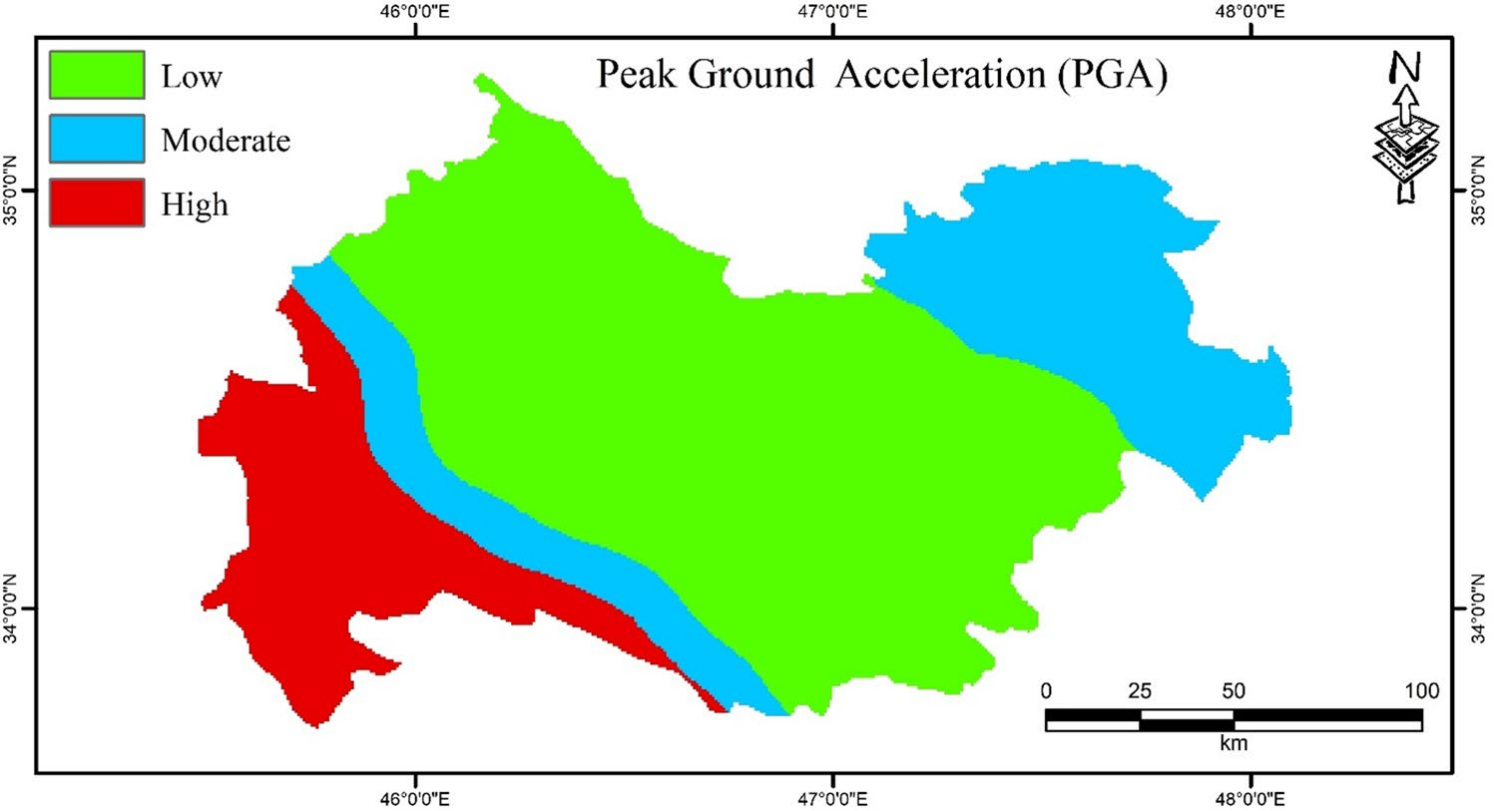
PGA map for the Kermanshah province using ArcGIS 10.3.1 software.

The PGA map for the Kermanshah Province is shown in Fig. 4. The PGA map was classified into three classes: low, moderate, and high, which cover 59%, 25%, and 16% of the province, respectively. The lowest class was observed in the central parts of the region, whereas the highest class was in the western part of the Kermanshah Province.

### Multi-hazards Map (MHM)

Maps of three hazards (i.e., earthquakes, floods, and landslides) were combined to generate a multi-hazard map using a combined tool in ArcGIS software. The multi-hazard map (MHM) was obtained as:

1 $${\text{MHM}} = {\text{SAPF}} + {\text{SAPL}} + {\text{PGA}}$$

**Figure 5:**
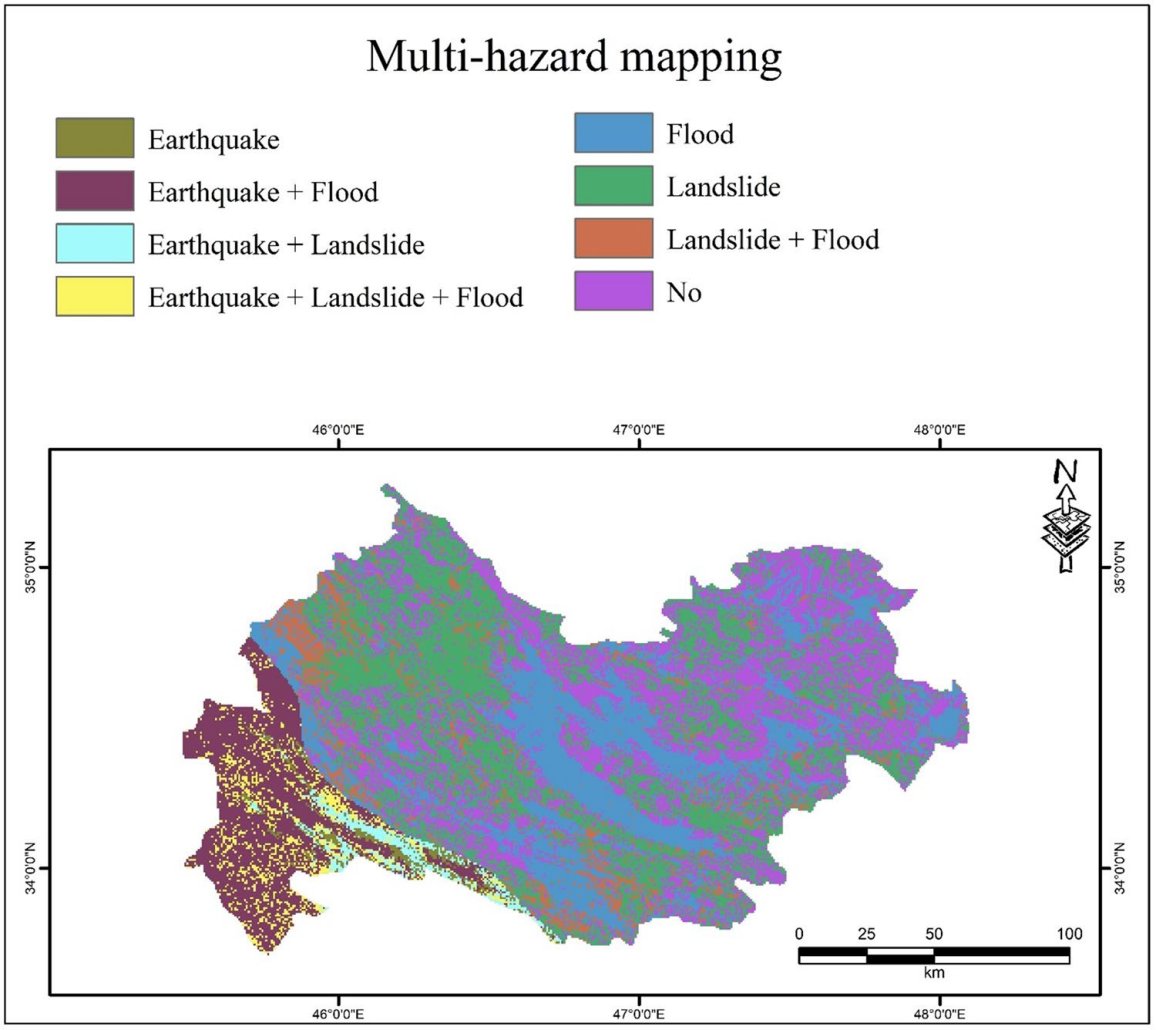
Multi-hazard map for the Kermanshah province using ArcGIS 10.3.1 software.

**Figure 6:**
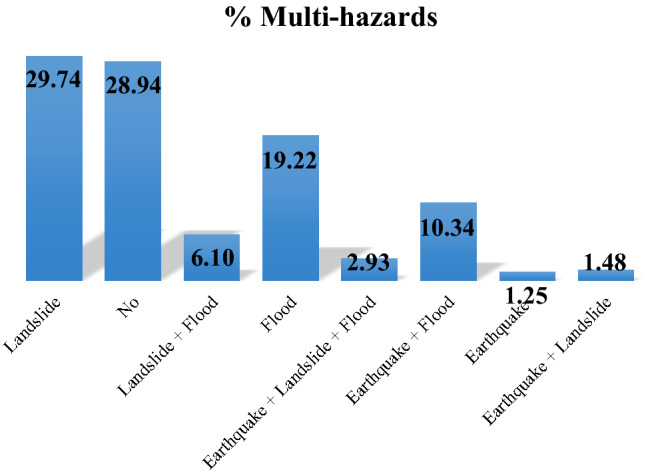
Percentage of areas of hazard classes.

Figure 5 shows the multi-hazard map for the Kermanshah province. The map was reclassified into eight classes. The multi-hazard map demonstrates that 2.93% of the area is faced with all three hazards, while 28.94% of the area is safe for these hazards. In addition, the distribution of other hazards indicates that 29.74%, 6.10%, 19.22%, 10.34%, 1.25%, and 1.48% of the Kermanshah province are affected by landslides, (landslide + flood), (flood), (earthquake + flood), earthquake, and (earthquake + landslide), respectively (Fig. 6).

### The advantages of natural multi-hazards studies

First, it should be noted that there are substantial differences in the natural hazards of earthquakes, floods and landslides. An earthquake is a sudden and rapid movement that is caused by breaking and moving rocks deep inside the earth. Moreover, a flood can be defined as an overflow of water from the natural range of a watercourse or body of water and or the accumulation of drainage water in areas that are not normally underwater. Landslide is also defined as the mass movement of rock, debris, and downward movement toward gravity under gravity, which causes the loss of one or more soil functions (https://www.recare-hub.eu/soil-threats/floods-and-landslides). Despite these substantial differences in these hazards, they can still be assessed together in a study and a comprehensive map can be prepared. In recent years, researchers' interest in studying multiple natural hazards has increased significantly^37,38^. Therefore, generating a multi-hazard map is of great importance for the integrated management of natural resources because it can help to reduce or even prevent economic and human losses. Natural hazards can have major effects and interact with each other. The mechanism of these interactions to come into being is different, and one may cause the occurrence or stimulation of another event^48^. Landslides, for example, can cause dangerous floods, especially when landslides in creeks are broken. Flooding also increases the possibility of landslides^49^. Liu et al.^48^ also stated that earthquakes cause landslides and storms cause floods. Temporary small-scale floods can significantly affect soil erosion. Floods on slopes are related to soil erosion and landslides in various ways, such as surface flow, sheet flow, return flow, and groundwater furrow. Moreover, the accompanying flood may destroy soil cavities and soil organisms that make up the soil structure. Therefore, the simultaneous study of several hazards in one study covered all gaps in individual studies. Multiple hazard assessments were performed to address the limitations of a single risk assessment.

### Advantages and disadvantages of the applied models

Each model has their own advantages and drawbacks. The advantages of the ANFIS algorithm are as follows: (1) the ability to process numerous inputs, (2) the ability to maintain the advantages of combining two fuzzy algorithms and an artificial neural network, and (3) a robust method^50^. However, its disadvantage is its sensitivity to overfitting; therefore, the training phase should be performed carefully. The best way to use this algorithm effectively is to use meta-heuristic techniques in the model training phase. The combination of learning-metaheuristic algorithms increase the predictive power and accuracy of the models. Therefore, two ensemble models, namely, SWARA-ANFIS-PSO and SWARA-ANFIS-GWO, were applied to assess the susceptibility to flood and landslide hazards. The advantages of the PSO algorithm are: (1) simplicity, (2) ease of operation, (3) no overlap, and (4) no calculation of mutation^51^. Moreover, the GWO algorithm also has the following advantages: (1) its simplicity, (2) flexibility, (3) robustness, (4) ease of operation, (5) low control parameters required, and (6) avoidance of local optimization^52^.

### Comparison with previous studies

Ensemble methods have dramatically increased because of their high efficiency in helping researchers conduct studies on natural hazards (landslides and floods). Moreover, ensemble models reduce the uncertainty of each algorithm and increase its reliability^53,54^. To ensure and support the results of this study, a comparison was made with previous studies related to the field of study. A combination of machine learning algorithm (ANFIS), ant colony optimization (ACOR), and differential evolution (DE) algorithms were used to study landslide susceptibility by Razavi-Termeh et al^55^. The results showed that the ANFIS-DE model was the suitable model (0.946). Wang et al.^36^ also reported that the combination of ANFIS-BBO (AUC = 0.9045) and ANFIS-ICA (0.9044) algorithms performed better than the standalone ANFIS model (AUC = 0.8407) in assessing flood susceptibility. The study by Mehrabi et al.^33^ also emphasized that the combination of GA-ANFIS, PSO-ANFIS, DE-ANFIS, and ACO-ANFIS models had proper performance for assessing landslide susceptibility. Arora et al.^56^ used ANFIS-GA, ANFIS-PSO and ANFIS-DE models to model flood susceptibility in a study in India. The ANFIS-GA model showed good results for determining flood susceptibility in the region. They concluded that the hybrid meta-heuristic and ANFIS models performed well and their performance evaluation with the AUC diagram confirmed the results. In the study of Arora et al.^56^, the AUC values are from 0.768 and 0.924. In this study, these AUC values were obtained from 0.880 to 0.936, which indicates the excellent performance of the models used in this study. Compared to another study using a combination of ANFIS and meta-heuristic algorithms, it was found that the performance of the present study is better than the study conducted by Hong et al.^57^ in which ANFIS-GA (0.8488) and ANFIS-DE (AUC = 0.8523) models were used to assess flood susceptibility. Moreover, compared with the study of Ahmadlou et al.^58^, it was observed that the results of the AUC values of the current study have higher accuracy in the study of flood susceptibility compared to ANFIS-BA and ANFIS-BBO models (0.703). Finally, it can be concluded that a combination of optimization and machine learning algorithms can be used to develop measures for reducing losses and sustainable management. In addition, the results of this study are practical and useful for assessing natural hazards.

### Limitations and future recommendations

This study had some limitations. Fundamental changes in factors affecting natural hazards are the main causes of hazards. However, knowledge about the factors affecting natural hazards is incomplete, and some of these unknown factors may still be present. Another limitation of this study was the limited data available. In future studies, it is suggested that more influencing factors should be used to assess hazards. It is also suggested that in future studies, infrastructure (e.g., schools, hospitals, etc.) should be considered for their optimal assessment and location.

## Conclusion

Designing a multi-hazard map for an area can reduce economic losses and mortality and result in integrated and organized management. The multi-hazard map in this study is a combination of three important hazards (i.e., earthquakes, floods, and landslides). In this regard, susceptibility maps of SAPL, SAGL, SAPF, and SAGF were generated for the Kermanshah province located in western Iran. The accuracy of the results was assessed using the ROC curve, RMSE and MSE values, and it was concluded that the SAPF and SAPL models had the best performance for both flood and landslide hazards. In addition, a PGA map was generated to assess earthquake hazards. Finally, a multi-hazard map was generated from the combination of SAPL, SAPF, and PGA. The results showed that the southwestern areas of Kermanshah Province are affected by all three hazards, covering 2.93% of the region, while 28.94% of the province is safe from these hazards. Landslides cover the largest area (29.74%) of the region. In the context of sustainable management, the results of this work can be applied as a practical tool for managers and experts in order to reduce losses. In addition to being able to be used to study several hazards together, these models can also be used individually in other contexts of natural disasters such as floods, landslides, erosions, forest fires, etc.

## Methodology

### Description of the study region

**Figure 7:**
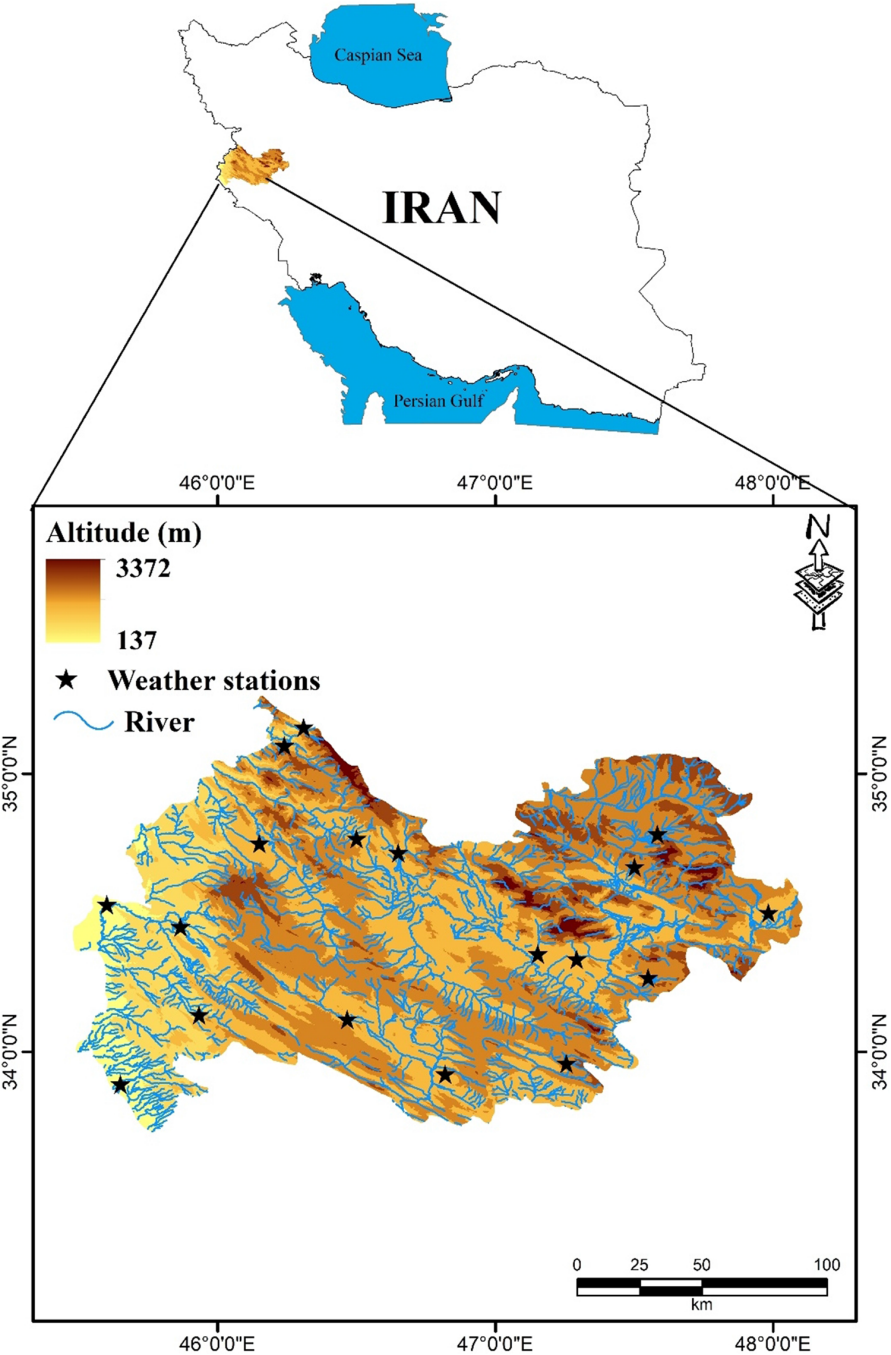
Location map of the Kermanshah province in Iran using ArcGIS 10.3.1 software.

The study area, Kermanshah Province, lies between 45°20 39″ to 48°01 58″ longitude and 33°37 08″ to 35°17 08″ latitude, with a total area of 24,650 km^2^ (Fig. 7). This province is the 17th province of Iran in terms of size. The study area is a mountainous area located between the Iranian plateau and the Mesopotamian Plain. The highest elevation in the region is 3,372 m. The climate of this province is classified as temperate or mountainous. Kermanshah Province is exposed to humid Mediterranean fronts, and snow and rain fall in colliding with the Zagros highlands. The mean annual temperature in Kermanshah Province was 15 °C. The coldest and warmest months were February and June, respectively. The average relative humidity in this area was over 40% (http://www.kermanshahmet.ir/met/amar).

### Hazards inventory mapping

This study considered three main hazards (i.e., earthquakes, floods, and landslides) in Kermanshah Province, Iran. The earthquake inventory map was obtained from the catalog of historical earthquakes in Iran provided by the International Institute of Earthquake Engineering and Seismology. The flooded areas were recognized using Google Earth Engine (GEE) and Sentinel-3 images considering the latest flood occurrences in the study area in 2019 and 2020, and they were then transformed into point data. The flood detection code was written on the GEE platform (https://earthengine.google.com). 617 flood points were extracted to prepare a flood inventory map in the ArcGIS platform. 70% of the points (432 points) and 30% of the points (185 points) were used for modeling and testing the models, respectively. Landslide location points were identified using historical data and field surveys by applying a global positioning system (GPS). 115 landslide points were used to prepare the landslide inventory map. 81 points (70%) was used for modeling and 34 points (30%) was used for testing the models. Most of the landslides occurred in the study area are of rotational and translational types.

### Multi-hazard influencing factors

Selecting factors that affect natural hazards is one of the first steps in hazard susceptibility maps. In this study, the influential factors were selected and classified based on previous research^59^. These influencing factors include altitude, slope aspect, slope degree, plan curvature, distance to river, distance to fault, distance to road, lithology, rainfall, and land use. The details and sources applied in the current study are listed in Table 5. All thematic maps were generated using ArcGIS 10.3. The details of the preparation of the influencing factors are as follows:

**Table 5:** Data and their sources used for susceptibility mapping of two hazards.

Data	Sources	Landslide influencing factor	Flood influencing factor
DEM (digital elevation model) (m)	ASTER (Global DEM) 30*30 m	×	×
Slope degree (°)	Extracted from DEM	×	×
Slope aspect	Extracted from DEM	×	×
Plan curvature	Extracted from DEM	×	×
Distance to roads (m)	National Cartographic Center	×	–
Distance to faults (m)	Geological Survey of Iran	×	–
Distance to rivers (m)	National Cartographic Center	×	×
Lithology	Geological Organization of Iran	×	×
Land use	https://code.earthengine.google.com/1775e5c262dc1194cf194a7597dd40bb (Ghorbanian et al., 2020)	×	×
Rainfall (mm)	Weather stations placed in the study area	–	×

**Figure 8:**
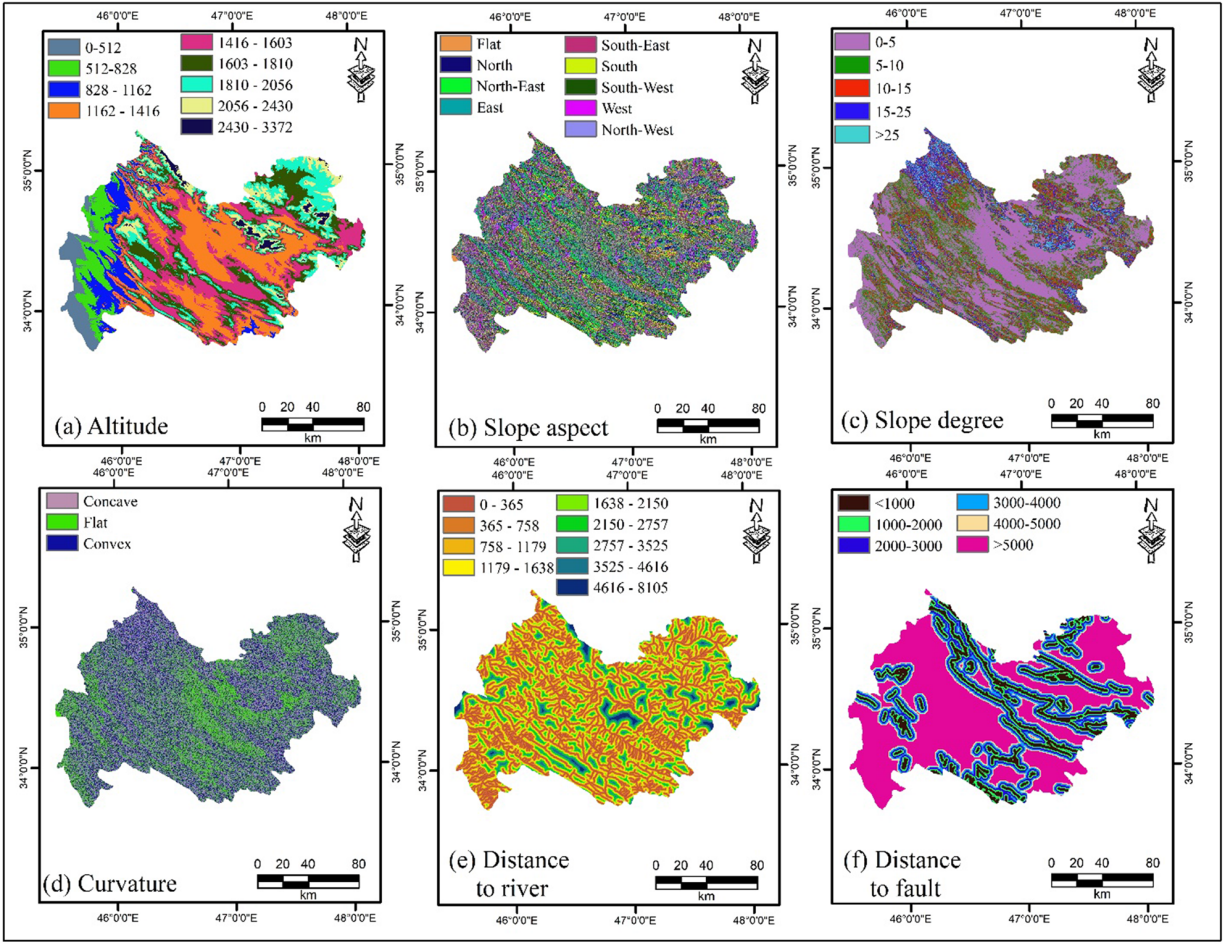

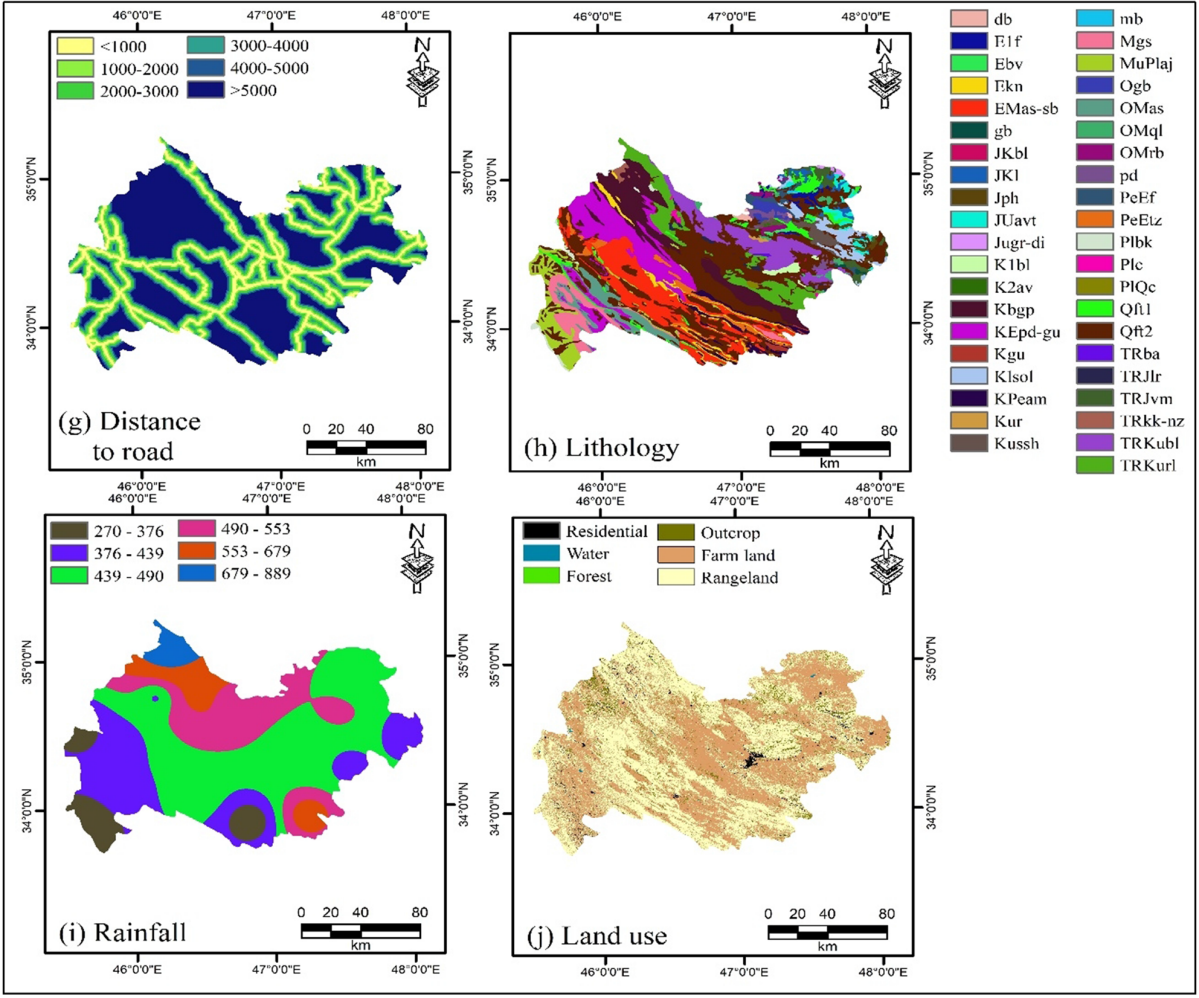
Map of multi-hazard influencing factors: (**a**) Altitude, (**b**) slope aspect, (**c**) slope degree, (**d**) curvature, (**e**) distance to river, (**f**) distance to fault, (**g**) distance to road, (**h**) lithology, (**i**) rainfall, and (**j**) land use using ArcGIS 10.3.1 software.

Altitude. Altitude is a useful influencing factor in natural hazard studies. In this study, the altitude factor was applied to create flood and landslide susceptibility maps. A basic digital elevation model (with resolution of 30 m × 30 m) was used to generate the altitude map (Fig. 8a).

Slope aspect. Slope aspect is a critical factor in natural disasters, which was used in this study to assess flood and landslide susceptibility. This factor is associated with variables such as rainfall, sunlight, and the morphology of an area, which is introduced as an effective parameter for the slope stability^34^. The slope aspect map for Kermanshah Province was classified into nine classes (Fig. 8b).

Slope degree. Another critical factor in natural hazard assessment is the degree of the slope. The slope degree map for the study region was classified into five classes. This layer was used to generate flood and landslide susceptibility maps (Fig. 8c).

Plan curvature. Plan curvature is a key factor in assessing the natural hazards of landslides and floods, which was considered in this study. This factor, which is the curvature of a flow and is shaped from the intersection of a vertical plane with the surface^22^ was generated for the study area. It was then classified into three classes (Fig. 8d).

Distance to river. Distance to the river is also one of the critical factors affecting the natural hazards of landslides and floods^17^. In areas with less distance to the nearest river, the probability of flooding increases^60^. River networks shape geomorphology, and rivers affect the support force on slopes^61^. The map of this factor was classified into nine classes (Fig. 8e).

Distance to fault. Faults are one of the factors influencing landslides and are used to generate landslide susceptibility maps. According to Conforti et al.^62^ as the distance to the faults decreases, the fracture rate and degree of rock weathering increase and the resistance decreases, resulting in an increase in the probability of landslide event. This map was classified into six classes (Fig. 8f).

Distance to road. The distance to the road is one of the factors influencing landslide assessment^22^ which was applied to produce a landslide susceptibility map. Road construction causes high slopes and disturbances in slope stability, resulting in multiple landslides^61^. The distance to the road map was classified into six classes (Fig. 8g).

Lithology. Lithology is one of the most critical factors influencing flood and landslide occurrence^36,46^, and was investigated in this study for flood and landslide hazards. The lithology of the area depends on different constructs and is determined by the type of rock that might affect landslide occurrence^63^. In addition, lithology plays a significant role in runoff control and surface infiltration because of its effect on soil permeability and porosity^46^. Geologically, the study area included a variety of units (Fig. 8h).

Rainfall. Rainfall is the primary cause of floods. Paul et al.^4^ also emphasized the strong correlation between flood and rainfall occurrences. As rainfall increases, the flood intensity also increases relatively. Long-term rainfall data (2001–2020) from meteorological stations located in Kermanshah Province were used to generate a flood susceptibility map. A flood map was obtained using the IDW interpolation method (Fig. 8i).

Land use. Land use plays a significant role in the natural hazard events. In the current work, land use was considered for both flood and landslide hazards. A variety of land uses have a major effect on the amount and number of floods^64^. This factor has a significant effect on the amount, frequency, and type of landslide, and can change the start threshold and accelerate it^65^. In total, six land-use groups were collected from Kermanshah Province through GEE^66^ (Fig. 8j).

### Multi-hazard spatial modeling

**Figure 9:**
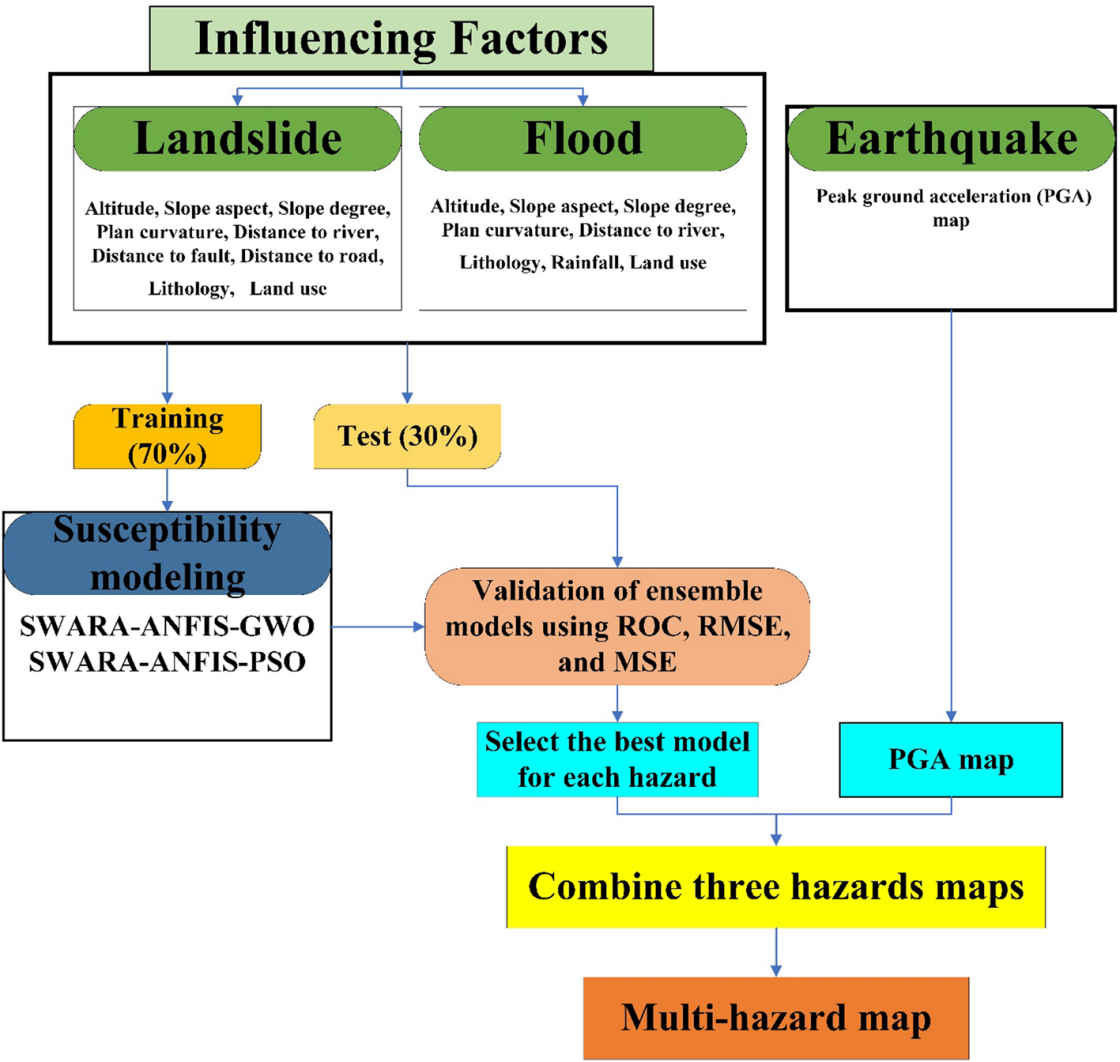
Methodological flowchart of the modeling steps for providing a multi-hazard map in the Kermanshah province.

Figure 9 shows the methodological flowchart of the modeling steps for providing a multi-hazard map in Kermanshah Province.

Multicollinearity diagnostic. Multilinearity is a statistical method used to determine the correlation of two or more independent variables (predictors) in regression analysis. The two methods of inflation coefficient of variance and tolerance are used to examine multicollinearity^67^. If the VIF is > 10 or tolerance is < 0.1, the multilinearity index of the independent variable is involved in regression analysis. In other words, there is the problem of multilinearity^67^.

### Step-wise weight assessment ratio analysis (SWARA) method

Keršulienė and Turskis^68^ introduced a decision-making model with the aim of weighting the criteria and sub-criteria. In this model, each expert based on his/her experience, knowledge, and information assigns a weight to each factor according to their importance. The steps in implementing this model are as follows. (1) Factor and sub-factor are selected. (2) Sub-factors are rated by experts based on their relative importance. The highest rate and the lowest rate are assigned to the most important sub-factor (first row) and the least important sub-factor (last row), respectively. For more information refer to Keršulienė and Turskis^68^.

### The ANFIS algorithm

ANFIS is an artificial neural network and fuzzy logic proposed by Jang in 1993^69^. This method can solve complex nonlinear problems. Takagi and Sugeno’s fuzzy method uses two if–then rules. Membership values are generated from the proper fuzzy sets using the membership function. The output of each node indicates the power of each rule^34,70^. See Zhang et al. ^69^ for more details.

### The PSO algorithm

PSO is proposed by Kennedy and Eberhart^71^ based on the random population and social behavior of birds in the wild^72^. This algorithm was used in the swarm intelligence (SI) algorithm group. The particles in this method show solutions to the problem, and the solutions (optimal solutions) are randomly identified by the vector. The velocity and position are the two main criteria for executing the algorithm. Each particle chooses the direction of movement based on the current position and the best position experienced between the particles. See Kennedy and Eberhart^71^ for more information.

### The GWO algorithm

The GWO algorithm is a technique inspired by the social life of grey wolves in wild^73^. In this algorithm, (α), (β), and (δ) wolves are the leaders of hunting, and the ω wolves follow them to identify the optimum solution. The GWO was modeled as follows: Social hierarchy, encircling prey, hunting, attacking prey, and searching the prey. Link available for more information: http: //www. alimirjalili.com/GWO. html.

### The ensemble models

In the current study, the GWO and PSO meta-heuristic methods were applied for training instead of the classic ANFIS model functions. The hybrid SWARA-ANFIS-PSO and SWARA-ANFIS-GWO algorithms were implemented using MATLAB software. In this regard, training and testing data for floods and landslides are required. Therefore, 70% of the data related to floods were defined for training hybrid models, and 30% were defined for testing models with code 1. The same number of non-flood points (70% training and 30% testing) was selected, and code 0 was assigned to them. In addition, for data on landslides, 70% of the training data (1) and 30% of the testing data (1) were selected, and the same number of non-landslide points (70% of training and 30% of testing) were defined with code 0. These points were then matched with the factors influencing natural hazards, and the corresponding values were obtained.

### Earthquake hazard map

Seismic hazard analysis methods can be used to predict the seismic behavior of a certain area. These methods study the probability of earthquake occurrences with different magnitudes in the study area using the seismic history of a region, historical seismic information, and seismotectonic investigation. Earthquake risk estimation methods include experimental-statistical, deterministic, and probabilistic methods^74^. In this study, a probabilistic method is used. In the probabilistic seismic hazard analysis method, all important earthquakes and springs with different distances to the site were considered, considering the occurrence probability of all events. The main assumption in the analysis of earthquake estimation using the probabilistic method is the randomness, and because these events are statistically independent, the time distribution function of these events is expressed by the Poisson distribution function. In addition to Poisson functions, there is another model called the iteration model, in which the occurrence of an event is related to the occurrence of previous events^75^. The steps in analyzing probabilistic earthquakes using the probabilistic method are: (1) identifying springs, (2) determining seismicity parameters, (3) selecting appropriate reduction relationships, and (4) calculating severe ground motion parameters^76^. Acceleration maps were estimated for a 475-year return period.

### Validation of ensemble models

In natural hazard studies, the accuracy of the results was verified after the implementation of the models^46^. The three criteria of RMSE, MSE, and ROC diagram were employed to estimate the accuracy of the ensemble models. RMSE and MSE are two standard statistical criteria for evaluating the accuracy of models^35^. These criteria were calculated as follows:

2 $$MSE = \frac{1}{N}\mathop \sum \limits_{i = 1}^{N} \left( {y_{i} - \hat{y}_{i} } \right)^{2}$$

3 $$RMSE = \sqrt {\frac{1}{N}\mathop \sum \limits_{i = 1}^{N} \left( {y_{i} - \hat{y}_{i} } \right)^{2} }$$

where $y_{i} { }$ and $\widehat{{y_{i} }}$ are the observed and predicted models.

This curve is designed based on two false-positive axes (x-axis) and true-positive values (y-axis)^62^. The values of the area under the curve are in the range [0.5–1], so that its numerical value determines the accuracy of the models used. The more the area under the curve move toward 1, the higher the accuracy of the model is, whereas the more the area under the curve moves toward 0.05, the lower the accuracy of the model is^62^. The ROC was obtained from Eq. (4).

4 $$ROC = \frac{\sum TP + \sum TN}{{P + N}}$$

### Multi-hazard mapping

A multi-hazard map of the total hazards of earthquakes, floods, and landslides was generated for the Kermanshah Province. First, SWARA-ANFIS-PSO-Flood (SAPF) and SWARA-ANFIS-PSO-Landslide (SAPL) susceptibility maps were generated and then classified into five classes. In the next step, a PGA map was generated for the earthquake hazard. The best selected models were again divided into two classes: 0 (very low, low, and moderate) and 1 (high and very high classes). In addition, the PGA map was reclassified into two classes: 0 (low and moderate) and 1 (high class). Finally, a multi-hazard map was generated by combining two-class maps of earthquakes, floods, and landslides for the Kermanshah province located in western Iran.

## Data Availability

The data used in this study are available for researchers upon request to the corresponding author for reasonable use in research.
